# Regulation of mTORC1 by the Rag GTPases

**DOI:** 10.1042/BST20210038

**Published:** 2023-03-16

**Authors:** Tshering D. Lama-Sherpa, Mi-Hyeon Jeong, Jenna L. Jewell

**Affiliations:** 1 Department of Molecular Biology, University of Texas Southwestern Medical Center, Dallas, TX 75390, U.S.A.; 2 Harold C. Simmons Comprehensive Cancer Center, University of Texas Southwestern Medical Center, Dallas, TX 75390, U.S.A.; 3 Hamon Center for Regenerative Science and Medicine, University of Texas Southwestern Medical Center, Dallas, TX 75390, U.S.A.

**Keywords:** amino acid sensing, mTORC1, Rag GTPases, signaling

## Abstract

The Rag GTPases are an evolutionarily conserved family that play a crucial role in amino acid sensing by the mammalian target of rapamycin complex 1 (mTORC1). mTORC1 is often referred to as the master regulator of cell growth. mTORC1 hyperactivation is observed in multiple diseases such as cancer, obesity, metabolic disorders, and neurodegeneration. The Rag GTPases sense amino acid levels and form heterodimers, where RagA or RagB binds to RagC or RagD, to recruit mTORC1 to the lysosome where it becomes activated. Here, we review amino acid signaling to mTORC1 through the Rag GTPases.

## Introduction

The ability of cells to sense extracellular and intracellular stimuli and respond appropriately is key to their survival. These stimuli include amino acids, growth factors, cellular energy levels, hormones, and stress [1]. Nutrient abundancy promotes anabolic processes like protein synthesis resulting in cell growth and proliferation, whereas nutrient deficiency initiates catabolic processes such as autophagy. The evolutionarily conserved mammalian target of rapamycin complex 1 (mTORC1) governs the cellular processes important for sensing nutrients. Dysregulation of mTORC1 is observed in several diseases such as cancer, obesity, metabolic diseases, diabetes, and neurodegeneration [2–4]. mTORC1 consists of three main subunits: mTOR, regulatory-associated protein of mTOR (Raptor), and mammalian lethal with Sec13 protein 8 (mLST8). mTOR is a Ser/Thr protein kinase that functions as a key catalytic subunit of two protein complexes mTORC1 and mTORC2. Further details on mTORC2 can be found elsewhere [5]. Raptor, recognizes and binds to mTORC1 substrates such as ribosomal protein S6 kinase beta 1 (p70S6K or S6K1) and eukaryotic translation initiation factor 4E-binding protein 1 (4EBP1) [6,7]. mLST8 positively regulates mTORC1 activity and stabilizes mTOR-Raptor binding [8]. Other mTORC1 components include proline-rich Akt substrate 40 kDa (PRAS40) and DEP-domain-containing mTOR-interacting protein (DEPTOR) that act as negative regulators [9–11]. Amino acids are one of the most potent activators of mTORC1, and the discovery of Rag GTPases has helped to identify the molecular mechanisms involved in amino acid sensing by mTORC1 [1,12]. The Rag GTPases act as a molecular switch coupling sensing of Leu, Arg, Met, Ala, His, Ser, Thr, and Val amino acids availability to ‘turn on’ or ‘turn off’ mTORC1 activity [12–14]. In this review, we have summarized the molecular mechanisms and identified components involved in amino acid sensing by mTORC1, specifically focusing on the Rag GTPases.

## Amino acid sensing by the Rag GTPases and mTORC1 regulation

**Figure 1.**
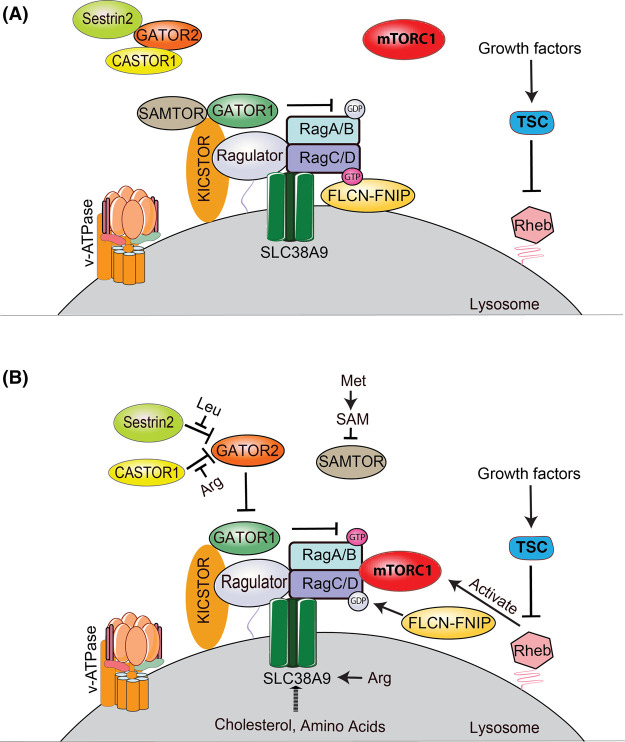
mTORC1 regulation by Rag GTPases. Amino acids and growth factors converge at the lysosome to activate mTORC1. Components implicated in amino acid sensing by mTORC1 via the Rag GTPases are Ragulator, v-ATPase, SLC38A9, KICSTOR, FLCN–FNIP, GATOR complexes (GATOR1 and GATOR2), Leu sensor Sestrin2, Arg sensor CASTOR1, and S-adenosylmethionine (SAM) sensor SAMTOR. TSC and Rheb regulate mTORC1 downstream of growth factors. (**A**) In absence of amino acids, GATOR1 exhibits GAP activity for RagA/B. Inactive Rag GTPases recruit TSC and FLCN–FNIP to the lysosomal surface where TSC's GAP activity prevents activation of Rheb. Sestrin2 and CASTOR1 interacts with GATOR2 and prevents GATOR2 from inhibiting GATOR1. (**B**) In presence of amino acids, folliculin and its associated proteins (FLCN–FNIP) acts as a GAP for RagC/D and GATOR2 inhibits GATOR1 GAP activity for RagA/B. The v-ATPase and SLC38A9 (arginine sensor) are required for mTORC1 activation at the lysosome. The active Rag GTPase heterodimer interacts with mTORC1 and recruits it to the lysosome subsequently promoting its activation by Rheb.

Two independent groups in 2008 identified that amino acids promote mTORC1 lysosome translocation and activation via the Rag GTPases [13,15]. There are four Rag genes in mammals, where RagA was thought to be functionally redundant with RagB, and RagC was thought to be functionally redundant with RagD [13,16,17]. However, recent studies reveal that Rag GTPases redundancy does not appear to be accurate [18,19]. Rags are in their ‘active’ form when GTP-bound RagA/B forms a complex with GDP-bound RagC/D. In contrast, in the ‘inactive form’, GDP-bound RagA/B complexes with GTP-bound RagC/D. The Rag GTPases ‘active’ and ‘inactive’ complex is controlled by the guanine nucleotide exchange factors (GEFs) and GTPase-activating proteins (GAPs) [20,21]. GAP toward Rags (GATOR) complexes acts upstream of Rag GTPases and regulates mTORC1 signaling. GATOR1 negatively regulates activation of Rag GTPase in absence of amino acids. GATOR2 inhibits GATOR1 in presence of amino acids [22,23]. Two important steps occur for amino acid-dependent mTORC1 activation: (1) In amino acid-rich conditions, the ‘active’ Rag GTPase heterodimer (GTP-bound RagA/B and GDP-bound RagC/D) recruit mTORC1 from an unknown location to the lysosome [24]. (2) Once at the lysosome mTORC1 encounters the small GTPase Ras homolog enriched in brain (Rheb). Rheb increases the catalytic activity of mTORC1 and results in mTORC1 activation [25,26]. Growth factors regulate the Tuberous sclerosis complex (TSC) which comprises hamartin (TSC1), tuberin (TSC2), and Tre3-Bub2-cdc16 1 domain family member 7 (TBC2D7) [27]. TSC acts as a GAP for Rheb and hence serves as a negative regulator of mTORC1 activity [28–30]. The Rag GTPases have also been reported to recruit TSC to the lysosome upon amino acid starvation, cellular stress, and growth factor restriction [31–33]. In presence of amino acid, the ‘active’ Rag GTPases bind to the mTORC1 subunit Raptor to promote mTORC1 lysosomal localization and activation. Conversely, when amino acids are absent, mTORC1 is not localized at the lysosome with the ‘inactive’ Rags and is instead associated with ADP-ribosylation factor GTPase-activating protein 1 (ArfGAP1) on unknown vesicles [34–38]. Thus, the Rag GTPases modulate mTORC1 activity based on amino acid availability. While most amino acids activate mTORC1 through Rag GTPases, Gln and Asn activate mTORC1 in a Rag-independent manner. This Rag-independent pathway requires the v-ATPase, Rheb, and the small GTPase ADP-ribosylation factor 1 (Arf1) [14,39–41]. Multiple components involved in the Rag GTPase pathway have been identified (Figure 1). We will summarize the Rag GTPase signaling pathway to mTORC1.

## Regulators of the Rag GTPases

### Ragulator

The Rag GTPases need a lysosomal localized anchor due to the lack of a lipid-anchoring motif [42]. The Ragulator complex, also known as a LAMTOR complex, functions as a scaffolding protein that tethers the Rags and mTORC1 to the lysosomal surface [42–44]. The Ragulator consists of five late endosomal/lysosomal adaptor, MAPK, and mTOR activator (LAMTOR) components: LAMTOR1 (also known as p18), LAMTOR2 (also known as p14), LAMTOR3 (also known as MP1; MEK-binding partner 1), LAMTOR4 (also known as C7orf59; chromosome 7 open reading frame 59), and LAMTOR5 (also known as HBXIP; hepatitis B virus X interacting protein) [44]. LAMTOR1 contains myristoylation and palmitoylation sites on the N-terminus, which are necessary for anchoring the Ragulator-Rag-mTORC1 complex to the lysosomal surface [45,46]. The Rag GTPase has two domains which include nucleotide-binding domains (NBDs) and C-terminal roadblock domains (CRD) [17,47,48]. The Ragulator binds the CRD of the Rag GTPase to tether it at the lysosomal surface. LAMTOR2, LAMTOR3, LAMTOR4, and LAMTOR5 also contain roadblock domains [20,49]. Additionally, the depletion of Ragulator components, LAMTOR1, LAMTOR2, and LAMTOR3 disrupts the localization of Rag GTPases and mTORC1 to the lysosome [12]. In addition to scaffolding, it has been reported that the Ragulator complex and SLC38A9 act as a non-canonical GEF for Rag GTPases in a vacuolar H^+^ — adenosine triphosphatase (v-ATPase)-dependent manner [20,47]. Overall, when amino acids are available the Ragulator complex promotes an ‘active’ Rag GTPase state to facilitate mTORC1 lysosomal localization and activation.

### v-ATPase

The v-ATPase is a highly conserved proton pump that hydrolyzes ATP. The v-ATPase is localized to intracellular organelles such as endosomes, lysosomes, and secretory vesicles [50]. The role of the v-ATPase is to pump protons across membranes acidifying organelles (such as the lysosome) and maintaining cytosolic pH [51,52]. The v-ATPase directly interacts with Ragulator and promotes its GEF activity for RagA/B resulting in mTORC1 activation [50,53,54]. Structurally, the v-ATPase is a multi-component complex consisting of two domains: the peripheral V_1_ domain, which contains eight subunits (A, B, C, D, E, F, G, and H) and hydrolyzes ATP, and the integral membrane domain V_0_, which contains six subunits (a, c, c’, c”, d, and e) [50,55]. The V_1_ domain interacts with the Ragulator depending on amino acid availability, while the V_0_ domain and Ragulator interaction remain unchanged by amino acid availability [20,56]. Amino acid sufficiency strengthens the Ragulator-v-ATPase interaction, whereas amino acid starvation weakens the interaction [56]. Pharmacological inhibition of the v-ATPase or v-ATPase knockdown alters the translocation of mTORC1 to the lysosomal surface even in presence of amino acids [56]. v-ATPase dysfunction prevents acidification which suppresses enzyme activity in the lysosomal lumen, leading to the disruption of amino acid flux from the lysosome to the cytoplasm [50].

### SLC38A9

The solute carrier transporter family regulates the transport of nutrients and metabolites across membranes [57]. Solute carrier family 38 member 9 (SLC38A9) has been shown to regulate amino acid signaling to mTORC1 [58]. Importantly, SLC38A9 is reported to facilitate the efflux of essential amino acids from the lysosome in an arginine-dependent fashion [59–61]. Arginine stimulates the interaction of the SLC38A9-Ragulator-Rag GTPases complex promoting mTORC1 signaling [62]. Other amino acids transporters like SLC36A1, SLC1A5, SLC3A2, and SLC7A5 have also been reported to regulate mTORC1 activity through the Rag GTPases [56,58]. In recent studies, the crystal structure of SLC38A9 was elucidated [63]. SLC38A9 is composed of 11 transmembrane helices, with a cytoplasmic N-terminus of 120 residues, and a C-terminus in the lysosomal lumen [63,64]. The cytosolic N-terminus domain of SLC38A9 interacts with the Ragulator (specifically LAMTOR1 and LAMTOR2) and the Rag GTPases [61]. The N-terminal region of SLC38A9 does not share sequence similarity with other SLC transporters and is unique to SLC38A9 [59]. Furthermore, SLC38A9 together with Ragulator has been reported to function as a non-canonical GEF for Rag GTPase resulting in mTORC1 activation [47]. Additionally, SLC38A9 is also important for cholesterol-driven mTORC1 activation independent of arginine [65].

### GATOR complexes

GAP toward Rags (GATOR) is made up of two subcomplexes called GATOR1 and GATOR2. GATOR 1 consists of 3 components: Disheveled egl-10 and pleckstrin domain-containing protein 5 (DEPDC5), nitrogen permease regulator 2-like protein (Nprl2), and nitrogen permease regulator 3-like protein (Nprl3). GATOR2 is composed of five components: meiosis regulator for oocyte development (Mios), WD repeat-containing domain (WDR24; WDR59), Seh1 like-nuceloporin (Seh1L), and Sec13 homolog (Sec13) [22]. During amino acid deprivation, GATOR1 displays GAP activity towards RagA/B, acting as a negative regulator of mTORC1 by promoting GTP hydrolysis of RagA/B [22,66]. In contrast, during amino acid abundance, GATOR2 inhibits GATOR1 and positively regulates mTORC1 [22,23]. Leucine sensor Sestrin2, and arginine sensor CASTOR1 binds GATOR2 and releases GATOR1. The exact mechanism of how GATOR2 inhibits GATOR1 is not yet understood. Recent studies have uncovered new insights into GATOR2 through structural studies [67,68]. Recently, RagA ubiquitination was found to promote its binding to GATOR1. RagA ubiquitination was mediated by E3 ubiquitin-protein ligase RNF152 leading to mTORC1 inhibition [69].

### Sestrin2

Cells have a variety of stress-inducible metabolic homeostasis regulators important for human disease. Among them, Sestrin2 has been identified as a leucine sensor [70]. In the absence of leucine, Sestin2 binds to GATOR2 resulting in mTORC1 inhibition. Leucine directly binds to Sestrin2 promoting the dissociation of Sestrin2–GATOR2 increasing mTORC1 activity. GATOR2 inhibits GATOR1 decreasing GATOR1's GAP activity toward RagA/B and subsequently promoting mTORC1 activity [71,72]. Leucine has a dissociation constant of 20 ± 5 µM, which is similar to physiologically cellular concentration levels of leucine [70]. Recent studies discovered that in *Drosophila*, Sestrin deficiency changes *Drosophila* adaptation to low-leucine diets and feeding behavior. Moreover, Sestrin deficiency fails to inhibit mTORC1 after leucine deprivation, resulting in impaired development and reduced lifespan in *Drosophila* [73].

### SAMTOR

S-adenosylmethionine (SAM) sensor for the mTORC1 (SAMTOR) is known as the SAM sensor and inhibits mTORC1 signaling. In the absence of SAM, SAMTOR interacts with GATOR1 and KICSTOR to inhibit mTORC1 [74,75]. SAM, which is synthesized from methionine and adenosine triphosphate by the methionine adenosyl transferase enzyme, binds to SAMTOR and disrupts SAMTOR binding with GATOR1 and KICSTOR. SAM has a dissociation constant of 7 µM like cellular concentrations of methionine. SAM binds to SAMTOR to signal methionine sufficiency for mTORC1 regulation [74]. For example, methionine starvation leads to the inhibition of mTORC1 activity in a SAMTOR-dependent manner. SAMTOR acts upstream of GATOR1 and KICKSTOR, which are both negative regulators of mTORC1 activity [74]. Additionally, a recent study has elucidated the structural and conformational changes in SAMTOR upon SAM binding [75].

### CASTOR1

Cellular arginine sensor for mTORC1 (CASTOR1) in parallel with SLC38A9 regulates arginine sensing to positively regulate mTORC1. CASTOR1 can exist as a homodimer or heterodimer with CASTOR2. When arginine is limiting CASTOR1 binds to and inhibits GATOR2 resulting in mTORC1 inhibition. Arginine directly binds to CASTOR1 which leads to disruption of the CASTOR1–GATOR2 increasing mTORC1 activity [76]. Arginine binds to CASTOR1 at a dissociation constant of ∼30 µM like physiological conditions. A recent study showed that CASTOR1 is a substrate for AKT, and E3 ubiquitin-protein ligase RNF167 detects the CASTOR1 phosphorylation by AKT resulting in its ubiquitination and subsequent degradation [77].

### KICSTOR

KICSTOR is a multi-protein complex consisting of kaptin, actin binding protein (KPTN), integrin alpha FG-GAP repeat containing 2 (ITFG2), chromosome 12 open reading frame 66 (C12orf66), and seizure threshold 2 homolog (SZT2) that recruits GATOR1 to the lysosomal surface to inhibit the Rag GTPase under amino acid starvation conditions [78]. The KICSTOR–GATOR1 complex interacts with the Rag GTPases and GATOR2 [78]. In addition, SZT2 deficiency results in mTORC1 lysosomal localization and activation even under nutrient deprivation [79]. Overall, KICSTOR negatively regulates mTORC1 signaling.

### FLCN–FNIP

The tumor suppressor Folliculin (FLCN) forms a complex with FLCN interacting proteins (FNIP) 1 or 2 and acts as a GAP for RagC/D, converting GTP-bound RagC/D to GDP-bound RagC/D. Mutations in the *FLCN* gene cause *Birt–Hogg–Dubé* syndrome. FLCN forms a complex with FNIP through its C-terminal domain and positively regulates mTORC1 [80,81]. During amino acid starvation, FLCN–FNIP localizes to the lysosomal surface and interacts with Rag GTPases. The FLCN–FNIP-Rag complex dissociates when amino acids are available, and FLCN–FNIP returns to the cytosol. Knockdown of FLCN inhibits mTOR lysosomal localization and activation in response to amino acid stimulation. Moreover, the N-terminal region of FLCN was determined to be important for the GAP activity toward RagC/D [81]. A recent study determined that GABA type A receptor-associated protein family (GABARAP) interacts with the FLCN–FNIP1 complex and sequesters it to the lysosomal membrane resulting in the disruption of the FLCN–FNIP1 complex's GAP activity toward RagC/D. GABARAP–FLCN–FNIP1 complex in turn impairs mTORC1-dependent TFEB phosphorylation to regulate selective autophagy such as xenophagy and mitophagy [82].

## Distinct functions of the Rag GTPases

RagA and RagB have ∼90% sequence similarity, whereas RagC and RagD have ∼80% sequence similarity in mammals [16]. In yeast, only one gene exists for RagA/B (Gtr1) or RagC/D (Gtr2). Hence, it has been traditionally thought that RagA and Rag B or RagC and RagD have redundant functions. Previous observations suggest that the Rags might have different functions based on different protein–protein interactions and structural differences. For example, human TSC2 strongly binds to RagA compared with RagB [33]. Mammalian leucyl-transfer RNA (tRNA) synthetase (LRS) has been reported to interact with GTP-bound RagD (not GTP-bound RagC) during leucine sufficiency, and LRS has been reported to have GAP activity only towards RagD and not RagC [83,84]. RagA and RagB share structural similarities but show differences in the N-terminus where RagB is extended by 33 amino acids compared with RagA [85]. RagC and RagD have differences in both their C- and N-terminal regions [16]. Furthermore, mice lacking RagA are embryonically lethal, whereas mice lacking RagB are viable indicating that the Rag paralogues may not be as functionally redundant as previously thought [86].

**Figure 2.**
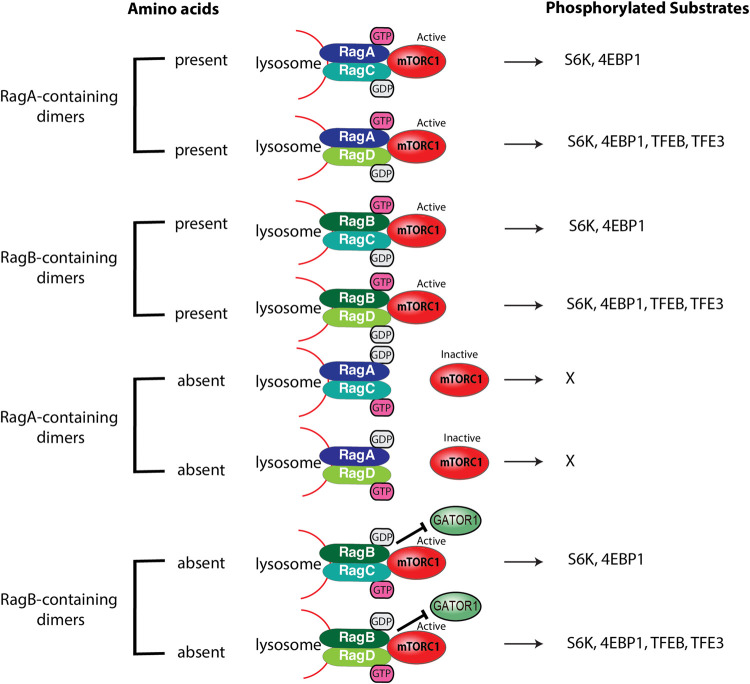
Different Rag GTPases heterodimers and mTORC1 substrates. The Rag paralogues form distinct heterodimer complexes to phosphorylate certain mTORC1 substrates.

Two recent studies show functional differences among the Rag GTPases. Their research showed that Rag paralogues have functionally diverse roles in amino acid signaling to mTORC1 [18,19]. Gollwitzer *et al.* [18] and colleagues showed that RagA and RagC/D heterodimers sense amino acid depletion and inactivate mTORC1, while RagB and RagC/D dimers retain mTORC1 at the lysosome even after amino acid starvation. Importantly, they found differences in mTORC1 substrate specificity among the different Rag GTPase complexes (Figure 2). RagA/B and RagD complex is important for TFEB phosphorylation by mTORC1, while both RagC/D dimers are important for the phosphorylation of S6K and 4EBP1 by mTORC1. Figlia and colleagues reported that RagB exists as two isoforms (short and long form), suggesting more diverse functions of Rags. The long form of RagB is specific to the brain and cardiac tissues while the short form of RagB is ubiquitously expressed in different tissues. Both the short and long forms of RagB were more resistant to decreased mTORC1 activity when amino acids are limiting, compared with RagA. The RagB short form inhibits the GAP activity of GATOR1 by binding to the DEPDC5 subunit, while the RagB long form inhibits GATOR1 GAP activity by binding NPRL2/3 subunits. Additionally, the Rag GTPases are differentially expressed in a wide variety of tissues, adding to the complexity of amino acid signaling to mTORC1 and the Rag GTPase heterodimers [19]. Different Rag isoforms and heterodimers may have unique tissue-specific physiological functions or binding partners. Thus, future studies will aid in identifying the specific mechanisms that lead to the activation or inhibition of mTORC1.

## Mutations in Rag GTPases and human diseases

Mutations in the Rag GTPase family have been associated with human disease [34]. Somatic mutations in *RRAGC* encoding for RagC have been observed in ∼17% of follicular lymphoma patients [87]. Mutations in *RRAGC* are predominantly missense and render a gain of function in RAGC leading to increased Rag-Raptor binding and constitutive activation of mTORC1 even when amino acids are limited [87]. Furthermore, another whole genome sequencing study in pediatric dilated cardiomyopathy patients revealed that Ser 75 was mutated to Tyr 75 in *RRAGC*, which leads to increased RagC GDP-loading and increased mTORC1 activity [88,89]. In support of these findings, a recent study with a hypomorphic mutant of RagC (RagC Glu 119 mutated to Leu 119) knock-in mice, displayed delayed follicular lymphomagenesis [90]. Similarly, another study found that a missense mutation in *RRAGD* impairs RAGD GTP-binding leading to a constitutively activated mTORC1 even in amino acid-depleted conditions in patients with Kidney Tubulopathy and Cardiomyopathy [91]. These findings demonstrate that mutations in the Rag GTPases can result in human disease. Additionally, in a cardiomyocyte RagA/B KO mouse model the mice exhibited increased hypertrophic cardiomyopathy and have lysosomal storage diseases [92]. Overall, the mutations in Rag GTPases have been implicated in follicular lymphoma, heart and kidney, and lysosomal storage disease.

## Perspectives

- The Rag GTPases sense amino acids and regulate mTORC1 lysosomal localization and activation.
- GTP-bound RagA/B heterodimerizes with GDP-bound RagC/D (‘active form’) and recruits mTORC1 to the lysosome. The nucleotide bound state of the Rag GTPases is modulated by GEFs and GAPs.
- New studies uncovered that the different Rag paralogues heterodimerize to perform unique functions. Thus, future studies should determine why these paralogues are differentially expressed in cells and tissues, and how it contributes to mTORC1 hyperactivation in human disease.

## Open Access

Open access for this article was enabled by the participation of University of Texas Southwestern Medical Center in an all-inclusive *Read & Publish* agreement with Portland Press and the Biochemical Society under a transformative agreement with Individual.

## Abbreviations

4EBP1: eIF4E-binding protein 1
AKAP: A-kinase anchoring protein
AKT: PI3K-Rac-alpha Ser/Thru-protein kinase
AMPK: 5′AMP-activated protein kinase
Arf1: adenosine diphosphate ribosylation factor 1
ArfGAP1: ADP-ribosylation factor GTPase-activating protein 1
cAMP: 3′,5′-cyclic adenosine monophosphate
CASTOR1: cellular arginine sensor for mTORC1
CDK1: cyclin-dependent kinase 1
DAG: diacylglycerol
DEPTOR: DEP-domain-containing mTOR-interacting protein
eIF4B: eukaryotic translation initiation factor 4B
ERK: extracellular signal-regulated kinase
FKBP12: prolyl-isomerase FK506-binding protein 12
GAP: GTPase-activating protein
GATOR1: GAP activity toward the Rags 1
GDP: guanosine diphosphate
GEF: guanine nucleotide exchange factor
GPCRs: G-protein coupled receptors
GPR137B: G-protein coupled receptor protein 137B
GTP: guanosine triphosphate
HEK293T: human embryonic kidney 293T
IKKβ: IκB kinase β
IP3: inositol 1,4,5-triphosphate
KICSTOR: KPTN, ITFG2, C12orf66, and SZT2
mLST8: mammalian lethal with Sec13 protein 8
MSN: medium spiny neuron
mTOR: mammalian target of rapamycin
mTORC1: mTOR complex 1
PDE: phosphodiesterase
PKA: protein kinase A
PRAS40: proline-rich Akt substrate 40 Kad
Raptor: regulatory protein associated with mTOR
REDD1: DNA damage and development 1
Rheb: Ras homolog enriched in brain
S6K1: p70 ribosomal S6 kinase 1
SLC38A9: solute carrier family 38 member 9
SREBP: sterol-responsive element-binding protein
TFEB: transcription factor EB
TOR: target of rapamycin
TSC: tuberous sclerosis complex
TSHR: thyroid stimulating hormone receptor
ULK1: Unc-51 like autophagy activating kinase 1
